# Psychometric Evaluation and Clinical Norms of a Dutch Version of the Body Uneasiness Test Among Individuals With Self‐Reported Eating Disorder Pathology

**DOI:** 10.1002/jclp.70143

**Published:** 2026-04-03

**Authors:** Lanaya M. van Uffelen, Edwin de Beurs, Bernou Melisse

**Affiliations:** ^1^ Department of Medical and Clinical Psychology Tilburg University Tilburg The Netherlands; ^2^ Department of Clinical Psychology Leiden University Leiden The Netherlands; ^3^ Stichting Arkin GGZ Amsterdam The Netherlands; ^4^ American Center for Psychiatry and Neurology, Al‐Manhal, Abu Dhabi Abu Dhabi United Arab Emirates; ^5^ Co‐Eur Utrecht The Netherlands; ^6^ Department of Clinical Psychology Utrecht University Utrecht The Netherlands

**Keywords:** body image, Body Uneasiness Test, factor structure, normative data, psychometric properties

## Abstract

**Introduction:**

The present study evaluates the psychometric properties and provides normative data for the Dutch Body Uneasiness Test. The instrument was administered to a clinical sample (*N* = 448) and a community‐based sample (*N* = 223). The Body Uneasiness Test was translated and back‐translated.

**Methods:**

Internal consistency, item‐rest correlation, test‐retest reliability, and concurrent validity were assessed. The Body Shape Questionnaire and the Eating Disorder Examination‐Shape concern subscale were used as reference measures. To establish criterion validity, a receiver‐operating‐characteristic curve‐analysis was performed using both groups as reference groups. Confirmatory factor analysis tested five‐ and eight‐factor structures. Norms (population‐based *T*‐scores and percentile scores) were established.

**Results:**

The psychometric properties were good, with the Body Uneasiness Test distinguishing well between individuals with and without eating disorder pathology (area‐under‐the‐curve value = 0.77–0.88). The Body Uneasiness Test‐A had a five‐factor structure, and the Body Uneasiness Test‐B had an eight‐factor structure. In accordance with other samples, clinical cut‐off for the Body Uneasiness Test A was 1.2.

**Conclusions:**

These results suggested that the Dutch Body Uneasiness Test is a useful tool for screening various aspects of body image in individuals with eating disorder pathology in research and in clinical practice.

## 1 Introduction

Body uneasiness falls under the broader concept of a negative body image defined as subjective negative thoughts and feelings about one's body and appearance (Holmqvist and Frisén 2010; Marano et al. 2007). Body uneasiness includes phenomena like worry, doubt and embarrassment, and is associated with psychological conditions, including anxiety, depression, low self‐esteem, and stress (Al‐Musharaf et al. 2022; Haddad et al. 2021; Kanj et al. 2024; Zeidan et al. 2019). Additionally, body uneasiness is associated with eating disorder pathology, and is suggested to be one of the core psychopathologic features of eating disorders (Fairburn et al. 2003). One of the maintaining mechanisms of body uneasiness is frequent body‐monitoring behaviors, such as pinching oneself, looking in the mirror, and comparing their body to others, to gain information about one's weight, shape, and size (Melisse and Arora 2024; Stefano et al. 2016). Such behaviors could perpetuate negative self‐assessment, leading to a cycle of increased body‐monitoring, and consequently, disordered eating. Therefore, reduction of a negative body image, including body uneasiness, is considered a key goal for treatment, especially given that greater body uneasiness at pre‐treatment is associated with poorer outcome and a higher relapse rate, whereas improvement in body image during treatment is associated with recovery (Cornelissen and Tovée 2021; Lee et al. 2018; Melisse and Dingemans 2025; Vall and Wade 2015).

It is critical to increase the identification of body uneasiness. The most commonly used self‐reported instruments to measure negative body image are the Body Shape Questionnaire (BSQ; Cooper et al. 1987; Melisse et al. 2024) and the shape concern subscale of the Eating Disorder Examination‐Questionnaire (EDE‐Q; Aardoom et al. 2012; Fairburn and Beglin 2008). There are, however, concerns regarding the factor structure of the EDE‐Q (Allen et al. 2011). This raises questions about the validity of the shape concern subscale. In addition, the BSQ assesses only one dimension of negative body image, namely body dissatisfaction (Marano et al. 2007), despite the broader spectrum of dissatisfaction, behaviors, and thoughts often reported by individuals with a disturbed body image (Cuzzolaro et al. 2006). The self‐reported Body Uneasiness Test (BUT; Cuzzolaro et al. 2006) is developed with the primary purpose of examining body uneasiness. The BUT examines in a first section body dissatisfaction, but also various aspects of negative body image, such as weight phobia, body image concerns, body avoidance, compulsive self‐monitoring and depersonalization (BUT‐A). Moreover, concerns towards specific body parts are addressed in a second section (BUT‐B). According to factor analysis, the BUT‐A has five subscales and the BUT‐B eight subscales. The English version of the BUT has good psychometric properties, primarily studied in samples with eating disorders and larger bodies or in Italy and Croatia (Cuzzolaro et al. 2006; Marano et al. 2007; Melisse et al. 2025; Pokrajac‐Bulian et al. 2015). Furthermore, the BUT is suggested to be a suitable instrument to measure body uneasiness across all eating disorders characterized by overvaluation of shape and weight (APA 2022). Compared to community samples, BUT scores are higher among women diagnosed with anorexia and bulimia nervosa (Cuzzolaro et al. 2006), individuals with binge‐eating disorder (Cuzzolaro et al. 2008), among transdiagnostic samples (Melisse et al. 2025), and among individuals with other eating disorder pathology (Aiello et al. 2022). Additionally, BUT scores are generally higher among individuals seeking weight loss treatment (Marano et al. 2007), and BUT scores are positively associated with Body Mass Index (BMI, kg/m^2^; Mento et al. 2017; Petta et al. 2016).

Examining the psychometric characteristics of the BUT in the Netherlands yields significant benefits for understanding and addressing body uneasiness within the Dutch context. Especially, availability of norms for clinical and community‐based samples, women and men and various age groups will facilitate more precise interpretation and better identification of individuals at risk or in need of treatment. Evidence from the original validation and subsequent work indicates that BUT scores differ by gender and age, underscoring the need for specific (Cuzzolaro et al. 2006; Marano et al. 2007; Melisse et al. 2025). The BUT offers a reliable and culturally relevant tool to assess body dissatisfaction, and additionally measure broader aspects of negative body image, such as avoidance and compulsive self‐monitoring, as well as inventory the specific body parts which cause the negativity (Cuzzolaro et al. 2006). This may lead to more accurate diagnostics, targeted interventions, improved treatment outcomes for eating disorders, and enhance knowledge into the cultural aspects on body uneasiness (Cornelissen and Tovée 2021; Túry et al. 2010). Furthermore, it can serve as a foundation for future research and enhanced tailored interventions.

Aim of the present study is to examine the psychometric properties (internal consistency, item‐rest correlation, test−retest reliability, concurrent validity, criterion validity, and factor structure) of the Dutch version of the BUT using data from respondents with self‐reported eating disorder pathology and respondents from the general population. Furthermore, norms will be provided in the form of percentile scores and normalized *T*‐scores.

## 2 Methods

### 2.1 Participants and Recruitment

The participants were recruited between December 2023 and July 2024. The clinical sample was recruited through various eating disorder clinics, as well as (mental) health‐care professionals, such as general practitioners and dieticians, and through patient initiatives. The community sample was recruited through social media. In addition, participants in both samples, could receive three study credits for study participation. Inclusion criteria for the clinical sample (*N* = 448) were: (a) diagnosed elsewhere with an eating disorder, (b) aged ≥ 16 years old, (c) sufficient proficiency in the Dutch language, and (d) web access. Inclusion criteria for the community sample (*N* = 223) were: (a) aged ≥ 16 years old, (b) sufficient proficiency in the Dutch language, and (c) web access.

### 2.2 Design and Procedure

The present validation study included cross‐sectional data from two convenience samples: one sample with self‐reported eating disorder pathology (clinical sample) and community‐based sample (community sample). Prior to the study, participants were informed about the purpose of the study and signed an informed consent statement, which was performed online through Qualtrics (Qualtrics 2024). Since participants were aged ≥ 16 years old parents did not have to sign the informed consent statement (World Medical 2001). Subsequently, demographic data were obtained, and eating disorder pathology and body image were assessed by self‐report. The BUT, the BSQ, and the Eating Disorder Examination Shape Concern subscale [EDE‐Q‐SC] were administered in a fixed order. Subsequently, participants were invited to sign up for a repeat assessment 1 week after, followed by a thank you note. After 1 week, self‐report instruments were readministered. The entire study was conducted on the web, anonymously through Qualtrics, which is ISO 27001/27017/27018 certified (Qualtrics 2024). The study was approved on 12‐12‐2023 by the ERB under file number TSB_RP1246.

### 2.3 Translation

To establish a Dutch version of the BUT (See Supporting Information S1: Table 1), the original English version was translated by two native speakers, one with specific eating disorder expertise, and by a certified translator without clinical specialization. The two Dutch translations were compared by a third translator with eating disorder expertise. Discrepancies among the two versions were discussed until consensus was established about the final version. This procedure was repeated by three new translators, who were blinded to the original English version of the BUT, in order to obtain a back translation (Swami and Barron 2019). The back‐translated version was compared to the English version and discrepancies were resolved by revising the Dutch translation. Subsequently, a brief pilot study was conducted among Dutch university students. They rated comprehension of all Dutch items on a 4‐point scale (1: I do not understand this at all; 4: This is completely clear). The results indicated that the translation was sufficiently understandable (mean comprehensiveness rating of all items: *M* = 3.4, SD = 0.2). No additional qualitative feedback on item wording was systematically collected, and no formal content validity indices were computed.

### 2.4 Materials

#### 2.4.1 BUT

The BUT (Cuzzolaro et al. 2006) is a self‐report questionnaire which measures various dimensions of negative body image in individuals with eating disorder pathology. This instrument consists of two sections: the BUT‐A (34 items; five subscales), which evaluates general body uneasiness, and the BUT‐B (37 items; eight subscales), which assesses discomfort with specific body parts and functions. Participants are required to respond on a 6‐point Likert scale from 0 (never) to 5 (always). Higher scores indicate greater body uneasiness. The BUT‐A Global Severity Index is the averaged score on all items (range 0−5). The total score of the BUT‐B consists of two parts, (i) the Positive Symptom Total (PST), operationalized as the sum of items scored higher than 0 (range 0−37), and (ii) the Positive Symptom Distress Index (PSDI), operationalized as the averaged total score on the PST items (range 0−5; Cuzzolaro et al. 2006). The original validation demonstrated adequate internal consistency (Cronbach's *α* > 0.70) and good test−retest reliability (*r* > 0.70) for the subscales (Cuzzolaro et al. 2006). Moreover, the original factor structure showed acceptable model (GAFI = 0.87−0.93; RMSEA = 0.8; Cuzzolaro et al. 2006).

#### 2.4.2 Body Shape Questionnaire

The Dutch version of the BSQ (Cooper et al. 1987; Melisse et al. 2024) is a self‐report questionnaire used to measure negative body image over the past 28 days. The BSQ has 34 items, answered on a 6‐point Likert scale from 1 (never) to 6 (always). The total score is the sum of the 34 items (range 34−204), with higher scores indicating more severe negative body image. The Dutch version of the BSQ is described elsewhere in detail (Melisse et al. 2024). The BSQ has high internal consistency (Cronbach's *α* = 0.81), good test‐retest reliability (*r* = 0.88) and a unidimensional factor structure (Cooper et al. 1987; Melisse et al. 2024; Melisse et al. 2022). Internal consistency in the present study is excellent (Cronbach's *α* = 0.97; McDonald's *ω* = 0.97). Given that the Dutch BSQ is only validated in a binge‐eating disorder sample (Melisse et al. 2024), to increase validity, the Dutch EDE‐Q‐SC is used for comparison as well, despite its questionable factor structure (Aardoom et al. 2012; Melisse et al. 2021).

#### 2.4.3 Eating Disorder Examination Questionnaire Shape Concern Subscale

The EDE‐Q‐SC (Aardoom et al. 2012; Fairburn and Beglin 2008) is the shape concern subscale of the EDE‐Q. The EDE‐Q is a self‐report questionnaire to screen for the presence or absence of eating disorder pathology over the past 28 days. Participants answer on a 6‐point Likert scale, with items running from 0 (no day at all) to 6 (every day/clearly). The total score on the EDE‐Q consists of the average score of all items (range 0−6). Higher scores indicate greater eating disorder pathology severity. The EDE‐Q‐SC consists of eight items measuring for example, the desire for a flat stomach, and dissatisfaction with body shape. Among the subscales of the EDE‐Q, the EDE‐Q‐SC has the highest internal consistency (Cronbach's *α* = 0.91) and adequate test‐retest reliability (*r* > 0.66) (Aardoom et al. 2012; Berg et al. 2012). Internal consistency in the present study was excellent (Cronbach's *α* = 0.91; McDonald's *ω* = 0.91).

#### 2.4.4 Demographics and Comorbid Psychopathology

Various demographics (age, gender, level of education, ethnicity, professional status, marital status, living situation), and comorbid psychopathology were administered through self‐report by a pre‐established list. Anthropometric data (weight and height) were collected as part of the EDE‐Q. Based on the anthropometric data, BMI was calculated in SPSS‐29 (IBM Corp 2024).

### 2.5 Data Analysis

Prior to the data analysis, participants who had > 5% missing data were removed from the dataset. The analyses were performed in SPSS‐27 (IBM Corp 2024), AMOS‐26 (Arbuckle 2019), *R* and *R* packages Lavaan, version 0.6‐5 (Rosseel 2012), the *R* RNOmni package version 1.0.1 (McCaw 2019), nls and nlstools (Wu and Estabrook 2016), and mirt (Chalmers et al. 2015). A significance level of *p* < 0.05 was maintained for all analyses. The results were reported in accordance with the Standards for Reporting of Diagnostic Accuracy Studies guidelines (Jérémie et al. 2022).

#### 2.5.1 Group Comparability

A Shapiro−Wilk test was administered to test the assumption of normality and Levene's test for the assumption of homogeneity of variances. The Shapiro−Wilk test was used for the assumption of normality. A visual inspection of a scatter plot was used for the assumption of linearity. Spearman's correlation was used if the assumption of normality or linearity was violated (Spearman 1961). When the assumption of homogeneity was violated, the test for unequal variance was used. Age, BMI, and scores on the self‐reports between both samples were compared with an independent *t*‐test. Group comparisons on gender and comorbid psychopathology were performed by chi‐square tests. Finally, comparisons on ethnicity, education, profession, marital status, living situation, and comorbid psychological disorder were tested using Mann−Whitney *U* tests.

#### 2.5.2 Reliability

Internal consistency was considered good when Cronbach's *α* and McDonald's *ɷ* were > 0.80 (MacDonald 1999). Item‐rest correlation was considered sufficient when the correlations were > 0.30 (Zijlmans et al. 2018). Test‐retest reliability was tested with an Intraclass Correlation Coefficient following recommendations by de Vet et al. (2006), and considered acceptable when *r* > 0.70 (Portney and Watkins 2009). An ICC value of 0.50−0.75 is considered moderate, while > 0.75 is considered good (Liljequist et al. 2019).

#### 2.5.3 Validity

Concurrent validity was determined by the correlation between the BUT‐A and BUT‐B scores with the BSQ global score as well as the EDE‐Q‐SC score. Criterion‐related validity or known‐groups validity was determined by comparing the mean scores on the BUT with a *t*‐test for independent samples. When the assumption of normality was violated, the Wilcoxon rank‐sum test was used (Wilcoxon et al. 1963). When the assumption of homogeneity was violated, the *t*‐value for unequal variance was used (Ramseyer and Tcheng 1973). A receiver operating characteristic (ROC) analysis and the area under the curve (AUC) were also calculated for scores on the BUT‐A and BUT‐B. The AUC indicated how well the model could differentiate between the presence or absence of clinically negative body image, with an outcome between 0.70 and 0.90 being an average score (Swets 1988).

#### 2.5.4 Clinical Cut‐Off

Based on gender clinical cut‐offs were established and their sensitivity and specificity ascertained. The sensitivity indicated the probability that the instrument signaled a positive test result when the individual actually had a clinical level of negative body image. The specificity indicates the probability that the instrument signaled a negative test result when the individual actually had no clinical level of negative body image. The optimal cut‐off score was determined using the Youden‐index (Nahm 2022), calculated by subtracting the false positive values from the sensitivity after which a score remained; *J*. The cut‐off score with the highest *J*‐value was deemed the most optimal.

#### 2.5.5 Factor Analysis

The factor structures of the BUT‐A and BUT‐B were examined using confirmatory factor analysis (CFA). The response options were considered ordered, and a polychoric correlations was used, the DWLS estimator with NLMINB as the optimization method, and reported robust fit indices. When the RMSEA was < 0.08 and the TLI and CFI were > 0.90 the model had a good fit (Awang 2012; Byrne 1994).

#### 2.5.6 Norms

Clinical norms were established in the form of normalized *T*‐scores for the population based sample and Percentile Rankorder (PR) scores for both samples. First, the influence of gender and age on scores was examined in order to assess the need of separate norms for genders. Cross‐walk tables and ‐formulas to transform raw scores into *T*‐scores and PR‐scores were obtained with the RankNorm command. First raw scores of the population‐based sample were normalized through the Rankit approach (Bliss et al. 1956; Ipsen and Jerne 1944). RankNorm yields percentile ranks that were converted to normalized *Z*‐scores using the probit function, which calculates the inverse of the cumulative distribution function (Solomon and Sawilowsky 2009). *Z*‐scores were converted to *T*‐scores by *T* = 10*Z + 50; see de Beurs and Cloutier (2024) for details.

## 3 Results

### 3.1 Participants

In the clinical sample *N* = 641 participants signed the consent form, of which, *n* = 193/641 (30.1%) participants were excluded because of missing data. Eventually, the clinical sample included *n* = 434/641 (67.7%) participants who completed the BUT, BSQ, and EDE‐Q‐SC. In the community sample, *N* = 268 participants signed the consent statement, of which, *n* = 45/268 (16.8%) participants were excluded because of missing data. Consequently, the community sample consisted of *n* = 217/268 (81.0%) participants who completed all self‐reports.

Key demographic data are depicted in Table 1. Both samples showed violated normality (SW test: *p*'s < 0.001) and homogeneity (Levene's test: *p* < 0.001). Both samples had a right‐skewed age distribution with an overrepresentation of lower ages, and the clinical sample (*M* = 22.6, SD = 6.8) was younger than the community sample (*M* = 30.4, SD = 13.8); *t*(276.2) = −7.83 *p* < 0.001. The clinical sample had a significantly lower BMI, included more women, lower educated individuals, more students and single individuals, lived more often with their parents, and reported more often comorbid psychopathology compared to the community sample.

**Table 1:** Demographics of two convenience samples: a clinical sample (*N* = 448) and the community‐based sample (*N* = 223).

	Clinical sample	Community sample	*p*
	*n* = 448	*n* = 223	
Age, *M* (SD)	22.7 (6.8)	30.4 (13.8)	< 0.001
BMI, *M* (SD)	22.7 (5.1)	23.8 (3.7)	0.002
Gender, *n* (%)			< 0.001
Women	411 (91.7%)	146 (65.5%)	
Men	26 (8.0%)	77 (34.5%)	
Other	1 (0.2%)	0 (0.0%)	
Highest level of education, *n* (%)			< 0.001
No education	2 (0.4%)	2 (0.9%)	
Primary school	3 (0.7%)	1 (0.4%)	
Lower education	2 (0.4%)	20 (9.0%)	
Senior general secondary education/university preparatory education	288 (64.3%)	61 (27.4%)	
Secondary education	96 (21.5%)	100 (44.9%)	
University	54 (12.1%)	39 (17.5%)	
Unknown	3 (0.7%)	0 (0.0%)	
Ethnicity, *n* (%)			0.127
Dutch	395 (88.2%)	213 (95.5%)	
Dutch Caribbean	2 (0.4%)	0 (0.0%)	
Europe, other	24 (5.4%)	4 (1.8%)	
Other	26 (5.7%)	6 (2.6%)	
Unknown	1 (0.2%)	0 (0.0%)	
Profession, *n* (%)			< 0.001
Student	376 (83.9%)	97 (43.5%)	
Employed	48 (10.7%)	113 (50.7%)	
Volunteer	6 (1.3%)	2 (0.9%)	
Unemployed	5 (1.1%)	3 (1.3%)	
Other	13 (2.9%)	8 (3.6%)	
Marital status, *n* (%)			< 0.001
Single	406 (90.6%)	176 (78.9%)	
Registered partnership	20 (4.5%)	10 (4.5%)	
Married	20 (4.5%)	30 (13.5%)	
Divorced	2 (0.4%)	7 (3.1%)	
Living situation, *n* (%)			< 0.001
Alone	73 (16.3%)	50 (22.4%)	
Together without offspring	61 (13.6%)	36 (16.1%)	
Together with offspring	32 (7.1%)	42 (18.8%)	
Student housing	113 (25.2%)	42 (18.8%)	
With parents	169 (37.7%)	53 (23.8%)	
Comorbid psychopathology *n* (%)			< 0.001
No	131 (29.2%)	154 (69.1%)	
Yes	317 (70.8%)	69 (30.9%)	
Body Uneasiness Test, *M* (SD)			
BUT‐A	2.05 (0.90)	0.74 (0.75)	
BUT‐B PST	22.37 (7.78)	12.43 (8.41)	
BUT‐B PSDI	2.24 (0.55)	1.71 (0.58)	
Body Shape Questionnaire, *M* (SD)	107.38 (35.08)	63.98 (28.79)	
Eating Disorder Examination‐Questionnaire Shape Concern subscale, *M* (SD)	3.46 (1.55)	1.66 (1.43)	

*Note: p* values for age and BMI were tested with an independent *t*‐test, gender, ethnicity, education, profession, marital status, living situation and comorbid diagnosis were tested using chi‐square tests.

Abbreviations: BMI = body mass index, *M* = mean, PSDI = positive symptom distress index, PST = positive symptom total, SD = standard deviation.

### 3.2 Assumptions

Normality was violated in both samples on all scores (SW test: *p*'s < 0.001−0.019). All scores in both samples were right‐skewed with overrepresentation of low scores, except for the BUT‐B PST, BSQ, and EDE‐Q‐SC in the clinical sample, which were left‐skewed with an overrepresentation of high scores. In addition to non‐normality, the assumption of homogeneity was violated in the BUT‐A (Levene's test: *p* < 0.001). Linearity was violated in both groups on all scores. Supporting Information S1: Table A1 and A2 in the supporting materials provide information of the frequency distribution of items scores and frequency of use of response options for BUT‐A and BUT‐B, respectively. Most items of the BUT‐A had a normal distribution, items that showed some positive skewness and kurtosis due to overrepresentation of low responses were 8, 13, 15, 16, 24, 25, and 29. For all these items > 50% of the participants opted for the lowest response option. Skewness and kurtosis were higher among the BUT‐B items. Here, items with high skewness and kurtosis were 6, 7, 8, 10, 11, 13, 14, 16, 17, 21, 30, 32, 33, and 35.

### 3.3 Reliability

Table 2 shows internal consistencies, mean item‐rest correlations and test‐retest correlations of the subscales. Internal consistency of the BUT‐A total score was excellent in the clinical sample (Cronbach's *α* = 0.96, McDonald's *ω* = 0.96), and in the community sample (Cronbach's *α* = 0.97, McDonald's *ω* = 0.97) (Nunnally 1978; Watkins 2017). Internal consistency of the BUT‐B total score was also excellent in both samples (clinical sample: Cronbach's *α* = 0.91, McDonald's *ω* = 0.91; community sample: Cronbach's *α* = 0.94, McDonald's *ω* = 0.95).

**Table 2:** Mean item rest correlation, internal consistency, and test−retest reliability of the subscales of the BUT in a clinical sample (*N* = 448).

	*M* IRC	*α*	*ω‐h*	ICC
BUT‐A general severity index	0.63	9	82	0.54
BUT‐A weight phobia	0.63	0.87	0.88	0.58
BUT‐A body image concern	0.72	0.92	0.92	0.57
BUT‐A avoidance	0.67	0.87	0.86	0.47
BUT‐A compulsive self‐monitoring	0.50	0.73	0.74	0.57
BUT‐A depersonalization	0.67	0.87	0.87	0.58
BUT‐B I	0.47	0.73	0.73	0.59
BUT‐B II	0.45	0.72	0.71	0.60
BUT‐B III	0.56	0.79	0.79	0.57
BUT‐B IV	0.41	0.66	0.66	0.58
BUT‐B V	0.49	0.73	0.72	0.58
BUT‐B VI	0.49	0.66	0.68	0.74
BUT‐B VII	0.19	0.32	‐a	0.61
BUT‐B VIII	0.44	0.68	0.67	0.58

Abbreviations: α = Cronbach's alfa, BUT = body uneasiness test, BUT‐B I = items 7 + 8 + 9 + 10 + 11 + 12, BUT‐B II = items 2 + 3 + 6 + 13 + 14 + 15, BUT‐B III = items 24 + 25 + 28 + 29 + 30, BUT‐B IV = items 1 + 21 + 31 + 32 + 33, BUT‐B V = items 19 + 20 + 22 + 23 + 26, BUT‐B VI = items 16 + 17 + 18, BUT‐B VII = items 4 + 5, BUT‐B VIII = items 27 + 34 + 35 + 36 + 37, ICC = Intraclass Correlation Coefficient (Agreement), M IRC = mean item rest correlation, ω‐h = McDonald's omega‐hierarchical, ρ = Spearman's rho.

^a^ Could not be computed due to only two items;

*p < 0.001.

In the clinical sample the item‐rest correlations of the BUT‐A were not adequate for item 1 (I spend a lot of time in front of the mirror: *r*ir = 0.16). All the other items had adequate item‐rest correlations in both samples (*r*ir's > 0.30). In the clinical sample, the item‐rest correlations of the BUT‐B were not adequate for Item 16 (mustache: *r*ir = 0.24). In addition, item‐rest correlations for item 1 (height: clinical sample: *r*ir = 0.24; community sample: *r*ir = 0.21) and item 17 (beard: clinical sample: *r*ir = 0.06; community sample: *r*ir = 0.15) were inadequate in both samples. For all other items of the BUT‐B item‐rest correlation were adequate in both samples (*r*ir's > 0.30). The full item‐rest correlations are depicted in the Supporting Information S1: Table A5.

One week test‐retest reliability was calculated in a subsample of the clinical sample (*n* = 286/436; 65,6%). Test‐retest reliability was moderate for the BUT‐A (ICC = 0.54) and the subscales of the BUT‐A and BUT‐B, as can be seen in Table 2. Of note, there were differences between the participants who did complete the self‐reports 1 week after compared to those who did not. They were older (Mean difference = 7.2, SD = 0.8, *p* < 0.001), had a lower BMI (Mean difference = 1.8, SD = 0.4, *p* < 0.001), women (*p* < 0.001) and were higher educated (z = −4.6, *p* < 0.001). There were no differences in ethnicity, profession, marital status, living situation, comorbid psychopathology, BUT‐A, BUT‐B, BSQ, and EDE‐Q scores.

### 3.4 Validity

Concurrent validity is presented in Table 3. In the clinical sample the BUT‐A had medium to high correlations with similar scales, the BUT‐B PST and the BUT‐B PSDI had small to medium correlations. In the community sample the BUT‐A had medium to high correlations with similar scales, the BUT‐B PST medium to high correlations, and the BUT‐B PSDI medium correlations.

**Table 3:** Concurrent validity of the BUT, EDE‐Q‐SC and BSQ (Spearmans rho).

	Clinical sample			Community sample		
		BUT‐B			BUT‐B	
	BUT‐A	PST	PSDI	BUT‐A	PST	PSDI
BUT‐B PST	0.55			0.82		
BUT‐B PSDI	0.67	0.32		0.59	0.45	
EDE‐Q‐SC	0.85	0.43	0.61	0.82	0.65	0.53
BSQ	0.87	0.51	0.62	0.89	0.77	0.54

*Note:* All correlations were significant (*p* < 0.001).

Abbreviations: BSQ = Body Shape Questionnaire, BUT = Body Uneasiness Test, EDE‐Q‐SC = Eating Disorder Examination Questionnaire Shape Concern subscale, PSDI = Positive Symptom Distress Index, PST = Positive Symptom Total.

Criterion validity was supported as the clinical sample had higher scores compared to the community sample on the BUT‐A (clinical sample: Mdn = 2.21, IQR = 0.75, community sample: Mdn = 1.60, IQR = 0.70; *W*_*s*_ = 35651.50, *z* = −15.69, *p* < 0.001, *r* = −0.61), BUT‐B PST (clinical sample: Mdn = 23.00, IQR = 12.25, community sample: Mdn = 9.00, IQR = 11.00; *W*_*s*_ = 39419.50, *z* = −13.85, *p* < 0.001, *r* = −0.42), BUT‐B PSDI (clinical sample: Mdn = 2.00, IQR = 1.26, community sample: Mdn = 0.53, IQR = 0.80; *W*_*s*_ = 38349.500, *z* = −10.63, *p* < 0.001, *r* = −0.54). Table 4 shows the Area Under the Curve (AUC) and suggested cut‐off scores for women and men separately, including sensitivity, specificity, and comparison to the original found cut‐off.

**Table 4:** Clinical norms and AUC value for the BUT by Gender.

Women	AUC	Sensitivity	Specificity	Estimated clinical cut‐off	CC	Above NC	Above OC
BUT‐A	0.85	0.84	0.75	1.16	335	83.5%	82.7%
BUT‐B PST	0.81	0.81	0.72	15.5	321	N/A	N/A
BUT‐B PSDI	0.74	0.74	0.66	1.89	293	N/A	N/A
Men	AUC	Sensitivity	Specificity	Estimated clinical cut‐off	CC	Above NC	Above OC
BUT‐A	0.91	0.92	0.87	0.99	33	91.7%	77.8%
BUT‐B PST	0.87	0.94	0.74	10.5	33	N/A	N/A
BUT‐B PSDI	0.77	0.72	0.72	1.80	25	N/A	N/A

Abbreviations: AUC = area under the curve, BUT = body uneasiness test, CC = correctly classified, NC = new cut‐off, N/A = not applicable, OC = original cut‐off, PST = positive symptom total, PSDI = positive symptom distress index.

### 3.5 Factor Structure

For the BUT‐A items the Kaiser–Meyer–Olkin (*KMO*) measure of sampling adequacy (MSA) was 0.97, and Bartlett's test of sphericity was significant (*c*²(561) = 20032.7, *p* < 0.001), supporting the suitability of the BUT‐A data for factor analysis. For the BUT‐A a single‐factor showed almost sufficient fit: *χ* ^2^(527) = 4265.06, *p* < 0.001, *χ* ^2^/df = 8.09, CFI = 0.942, TLI = 0.938, SRMR = 0.063, RMSEA = 0.104 [0.101−0.107]. However, the fit of the original 5‐factor model showed better fit: *χ* ^2^(517) = 2135.62, *p* < 0.001, *χ* ^2^/df = 6.07, CFI = 0.959, TLI = 0.956, SRMR = 0.052, RMSEA = 0.088 [0.085−0.091]. Modification indices revealed further improved fit when allowing for correlation between item pairs 28 and 29 “feeling detached from my body” and “feeling that my body does not belong to me” and item pairs 5 and 19 “When I undress, I avoid looking at myself” and “I avoid mirrors”; furthermore, allowing for cross loading of item 2 on CSM, item 2 on Weight Phobia, item 29 on Weight Phobia, item 29 on Body Image Concerns and item 1 on Depersonalization improved fit further. The results for this modified 5‐factor model are *χ* ^2^(510) = 2084.45, *p* < 0.001, *χ* ^2^/df = 4.48 CFI = 0.972, TLI = 0.970, SRMR = 0.042, RMSEA = 0.073 [0.070−0.076]. Convergent validity of this model was supported: Average Variance Extracted (AVE) > 50 for all factors (WP = 0.65; BI = 0.75; AVO = 0.74; CSM = 0.58; DEPE = 0.76). The discriminant validity was assessed by the Heterotrait‐ Monotrait ratio. Three of the ten ratio's were 0.90 or higher (the usual cut‐off for HTMT), indicating insufficient “separateness” of the factors (Weight phobia−Body image concerns = 0.96; Weight phobia−Compulsive Self monitoring = 0.93; Avoidance ‐ Body image concern = 0.90). Supporting Information S1: Table B1 in the Supporting Information Materials shows the loading of items on their primary factor.

For the BUT‐B items the Kaiser–Meyer–Olkin (*KMO*) measure of sampling adequacy (*MSA*) was 0.93 (with a low value for item 17; *MSA* = 0.59), and Bartlett's test of sphericity was significant (*c²*(666) = 10992.6, *p* < 0.001), supporting the suitability of the BUT‐B items for factor analysis, as well. CFA indicated that, a single‐factor model had insufficient fit: *χ* ^2^(629) = 3601.79, *p* < 0.001, *χ* ^2^/df = 5.73, CFI = 0.869, TLI= 0.923, SRMR = 0.083, RMSEA = 0.085 [0.083−0.088]. The fit for the 8‐factor model was better: *χ* ^2^(601) = 2180.37, *p* < 0.001, *χ* ^2^/df = 3.63, CFI = 0.930, TLI = 0.923, SRMR = 0.067, RMSEA = 0.064 [0.061−0.066]. Modification indices revealed improved fit when allowing for correlation between items 10 and 11 (“lips” and “mouth”), items 2 and 3 (“shape of head” and “shape of face”), and items 34 and 36 (“odor” and “sweat”). The results for this 8‐factor model are *χ* ^2^(598) = 1848.09, *p* < 0.001, *χ* ^2^/df = 4.64, CFI = 0.973, TLI = 0.970, SRMR = 0.042, RMSEA = 0.075 [0.071−0.078]. Convergent validity of this model was partially supported: AVE > 50 for five out of eight factors (Mouth = 0.55; Face = 0.53; Abdomen = 0.64; Extremities = 0.45; Upper body = 0.54; Moustache/hairs = 0.53; Skin = 0.36; Blushing = 0.50). The discriminant validity was assessed by the Heterotrait‐Monotrait ratio. Only one of the 28 ratio's was too high (> 0.90, the usual cut‐off for HTMT: Abdomen/lower body‐Extremities = 0.92), indicating overall sufficient “separateness” of these factors. Table SB2 in the supporting materials shows the loading of items on their primary factor.

### 3.6 Population‐Based Norms

Table SA6 in the supporting materials presents means for both genders and for three age groups. Generally, men had lower scores than women. For instance, on the GSI score men (*n* = 112) had a mean score of *M* = 0.84 (SD = 0.77) and women (*n* = 541) *M* = 1.77 (SD = 1.04). According to a *t*‐test (two‐sided) there was a significant difference between men and women; *t*(203.3) = −10.87; *p* = 0.0000; Cohen's *d* = 0.93 [0.72, 1.14]. Regarding age, there was a significant negative correlation between age and GSI score (*r* = −0.18, *p* < 0.0001). The community sample was divided in three age groups and Table SA7 in the supporting materials presents mean scores on the BUT‐A and BUT‐B scales. Generally, scores diminished with age. As can be seen in Table SA7, the mean BUT‐A score for group 16‐20 (*n* = 246) was *M* = 1.84 (SD = 0.88), for group 21‐23 (*n* = 192) *M* = 1.44 (SD = 1) and for group 24‐72 (*n* = 216) *M* = 1.5 (SD = 1.23). ANOVA for age group revealed a difference between the groups *F*(2, 409.32) = 11.75; *p* < 0.001; *Eta* ^*2*^ = 0.030; according to Tukey's HSD group 16−20 and 21−23 differed (Cohen's *d* = 0.43, 95% CI [0.24−0.62]) and group 16−20 and 24−72 differed (Cohen's *d* = 0.32, 95% CI [0.14−0.51]), a small to medium effect size.

Supporting SC Tables present a cross‐walk table, irrespective of gender or age group, with all possible raw scores and corresponding population‐based *T*‐scores as well as population‐ and clinical PR‐scores. Gender specific and age group specific cross‐walk tables and ‐formulas are provided in the Supporting Information Materials also.

## 4 Discussion

The present study is the first examining the psychometric properties of a Dutch version of the BUT, which adequately measured body uneasiness in the Netherlands. The BUT is a reliable and valid instrument, and well able to discriminate between a clinical and a community‐based sample. Subsequently, internal consistency, test‐retest reliability, concurrent, and criterion validity were good. The original five and eight factor models were confirmed for the BUT‐A and BUT‐B, respectively. Discriminant validity of the BUT‐A factors was not fully supported, suggesting that a higher order factor structure may be considered as well. Finally, convergent validity of the BUT‐B was not fully supported as not all scales explained sufficient variance of their items.

The BUT consistently showed to be a valid and reliable instrument across multiple European countries (Cuzzolaro et al. 2006; Marano et al. 2007; Pokrajac‐Bulian et al. 2015). The present findings emphasize the strong psychometric properties previously identified and provide further evidence of the BUT as a widely applicable instrument that could be used across diverse populations, including individuals with eating disorders, those with high weight (BMI > 25, and BMI > 30), and those considering cosmetic or bariatric surgery. Additionally, the BUT can serve as a valuable tool in clinical practice, contributing to diagnostics, treatment planning, and assessing the effectiveness of interventions targeting body uneasiness. In scientific research, the BUT offers opportunities to explore the complexity of body uneasiness and its relationship with other psychological and behavioral variables (Al‐Musharaf et al. 2022; Haddad et al. 2021; Kanj et al. 2024; Zeidan et al. 2019).

The findings of the current study align with prior work in clinical eating disorder samples, which demonstrated elevated BUT scores and strong associations with measures of eating disorder pathology (Aiello et al. 2022; Cuzzolaro et al. 2006; Melisse et al. 2025). The current sample showed strong psychometric properties for the BUT. This finding adds to previous evidence that the measure reliably captures the multidimensional nature of body uneasiness among individuals with eating disorders. Although the BUT is most commonly applied in the context of eating disorders, growing evidence suggest that body uneasiness represents a broader transdiagnostic construct. The BUT has been widely applied in studies involving, among others, individuals with gender dysphoria (Fisher et al. 2014; Turan et al. 2024), cancer patients and survivors (Verri et al. 2024; Zucchetti et al. 2017), postpartum females (Mento et al. 2017; Zanardo et al. 2016), and in the context of ageing (Bellard et al. 2020). Collectively, these findings support the relevance of the BUT across a wide range of populations.

There is a lack of studies examining clinical cut‐offs for both subscales. The present study found a cut‐off of 1.16 for women for the BUT‐A, which is similar to the 1.20 cut‐off in Mediterranean samples, with the difference being smaller than one standard deviation from the mean of the various samples (Capoccia et al. 2015; Imperatori et al. 2019; Segura‐García et al. 2010). It is not clear if this original cut‐off score is based on gender, but is notably higher than the present found cut‐off score for men. This could be due to the relatively low percentage of men in the current clinical sample. However, it should be noted that in the present study, the clinical cut‐offs were determined based on a sample who were diagnosed elsewhere with an eating disorder. Participants eating disorder was not confirmed by a diagnostic interview in the present study. Nonetheless, the clinical sample was recruited through various eating disorder clinics and there were significant differences in BUT scores between both samples. Furthermore, the present study is the first to tentatively offer clinical cut‐offs by gender and of the BUT‐B, which could provide more insights into which body parts could be overvalued by individuals with eating disorder pathology.

The present study has several strengths. It is the first to examine the properties of the Dutch BUT in a convenience sample of individuals eating disorder pathology, and to compare this to a community‐based convenience sample. Various aspects of validity, reliability, and the factor structure were examined. The clinical sample was sizable, the community sample was smaller, and additional research on population norms seems warranted. The present study contributed to the assessment and knowledge of a broader range of negative body image, such as compulsive self‐monitoring and depersonalization, whereas other instruments focus on only one of these dimensions.

There are also limitations to the present study. A major limitation was that the eating disorder diagnoses in the clinical sample were not confirmed. No structured diagnostic interview was performed, and consequently, specific types of eating disorder pathology were not specified in the present study. In addition, the group‐comparability showed differences on key demographics, with the clinical sample reflecting individuals with eating disorder pathology (Pike et al. 2013). Some of the differences between the samples could have influenced the results. For instance, it is suggested that body appearance becomes less important with increasing age (Tiggemann and Kuring 2004), which could explain the lower scores in the community sample. In addition, since the community sample was self‐selected, there was a risk that respondents with a heightened interest in eating disorders or related issues were overrepresented in the sample (Melisse et al. 2021). This could have led to inflated scores on BUT compared to results obtained in a more representative population. Moreover, comorbid diagnostic information from both samples in the present study relied on self‐reports. Furthermore, there was selective attrition at retest, as only a subset of participants completed the self‐reports 1 week later. These participants were significantly older, more often women, had a lower BMI, and were more highly educated. This may have biased the test‐retest reliability estimates, especially given prior findings that men and individuals with lower cognitive ability were more likely to drop out in eating disorder populations (Melisse et al. 2025; Melisse et al. 2022). Another limitation is that, during the translation and pilot testing process, formal content validity indices were not computed, and detailed pilot sample characteristics were not collected. This limits the extent to which the content validity and semantic equivalence of the Dutch BUT can be evaluated in accordance with current best‐practice guidelines. Several recommendations can be made for future research in the validity of the BUT. Firstly, to screen for individuals at high‐risk (a score exceeding a clinical cut‐off) for clinical levels of body uneasiness, the present results of the BUT should be re‐examined using a sample with confirmed eating disorders by a structured interview. In addition, it's recommended to recollect data in a representative sample from the general population stratified on a number of key variables that appear to influence BUT scores, such as age and gender. Furthermore, it would be useful to examine different age groups since the importance of body appearance is suggested to decrease with age (Melisse et al. 2025). Moreover, collection of longitudinal data and examination of sensitivity to change would facilitate future use of the BUT to evaluate treatment outcomes, in the current study it couldn't be assessed since post‐treatment data were not available. Additionally, it's recommended that the items of the BUT‐B about facial hair are only administered to men. Finally, cross cultural and language examinations is suggested to assess if the BUT has the same underlying construct across language versions (Melisse et al. 2025).

## 5 Conclusions

In conclusion, the psychometric properties of the Dutch version of the BUT appeared good, as evidenced by support for the factor structure, good reliability indices, and convergent and known group validity indices. By extending the validation of the BUT to the Netherlands, present study strengthens the evidence base for its cross‐cultural applicability and emphasizes the importance of assessing body uneasiness as a multidimensional construct. Given the broad coverage of body‐related distress, the BUT offers clinicians and researchers a useful screening and measurement tool to capture aspects of body uneasiness that extend beyond appearance concerns alone. However, present study highlights the need for continued validation in diagnostically well‐defined and representative samples.

## Author Contributions

The manuscript has been written by Lanaya M. van Uffelen in collaboration with Bernou Melisse, and Edwin de Beurs.

## Funding

The authors have nothing to report.

## Ethics Statement

The study was approved on 12‐12‐2023 by the ERB of Tilburg University under file number TSB_RP1246.

## Consent

The authors have nothing to report.

## Conflicts of Interest

The authors declare no conflicts of interest.

## Supporting information

Body Uneasiness Test Supplementary materials.

## Data Availability

Upon reasonable request with B.M.
