# Supplementary material for: Psychometric Evaluation and Clinical Norms of a Dutch Version of the Body Uneasiness Test Among Individuals With Self‐Reported Eating Disorder Pathology
Source: J Clin Psychol. 2026 Apr 3;82(8):1177–87. doi: 10.1002/jclp.70143 (PMC13341028; doi:10.1002/jclp.70143)
Supplement: Supplementary file 1 — Body Uneasiness Test Supplementary materials. [file JCLP-82-1177-s001.docx]

**Supplementary Table S1**

*The Dutch Body Uneasiness Test*.

**BUT-A**

**Kruis met een X het antwoord aan dat uw ervaring op dit moment het best weergeeft**

Antwoordopties: nooit (0), zelden(1), soms(2), vaak(3), heel vaak(4), altijd(5).

1. Ik spendeer veel tijd voor de spiegel
2. Ik vertrouw niet op mijn uiterlijk: ik ben bang dat het plotseling zal veranderen
3. Ik vind de kleren leuk die mijn lichaam verbergen
4. Ik besteed veel tijd aan het denken over een aantal gebreken van mijn fysieke verschijning
5. Wanneer ik mij uitkleed, vermijd ik het om naar mijzelf te kijken
6. Ik denk dat mijn leven significant zou veranderen als ik een aantal van mijn esthetische gebreken zou kunnen corrigeren
7. Met anderen eten bezorgt mij angst
8. De gedachte aan enkele gebreken van mijn lichaam kwelt me zo erg dat het me verhindert met anderen te zijn
9. Ik ben doodsbang om aan te komen
10. Ik maak gedetailleerde vergelijkingen tussen mijn verschijning en die van anderen
11. Wanneer ik naar mezelf begin te kijken vind ik het moeilijk om te stoppen
12. Ik zou alles doen om enkele delen van mijn lichaam te veranderen
13. Ik blijf thuis en vermijd dat anderen mij zien
14. Ik schaam me voor de fysieke behoeften van mijn lichaam
15. Ik voel dat ik uitgelachen word om mijn uiterlijk
16. De gedachte aan enkele gebreken van mijn lichaam kwelt me zo erg dat het me verhindert te studeren of werken
17. Ik kijk in de spiegel voor een beeld van mijzelf waar ik tevreden over ben en ik blijf zoeken totdat ik er zeker van ben dat ik het gevonden heb
18. Ik heb het gevoel dat ik dikker ben dan anderen mij vertellen
19. Ik vermijd spiegels
20. Ik heb de impressie dat mijn beeld altijd verschillend is
21. Ik zou graag een dun en beenachtig lichaam willen hebben
22. Ik ben ontevreden over mijn uiterlijk
23. Mijn fysieke uiterlijk is teleurstellend vergeleken met mijn ideaalbeeld
24. Ik zou graag plastische chirurgie willen ondergaan
25. Ik kan het idee niet uitstaan om te leven met het uiterlijk dat ik heb
26. Ik kijk naar mezelf in de spiegel en heb een gevoel van onbehagen en vreemdheid
27. Ik ben bang dat mijn lichaam zal veranderen tegen mijn wil in, op een manier die ik niet leuk vind
28. Ik voel me vervreemd van mijn lichaam
29. Ik heb het gevoel dat mijn lichaam niet van mij is
30. De gedachte aan bepaalde gebreken van mijn lichaam kwelt me zo erg dat het me verhindert een seksueel leven te hebben
31. Ik observeer mezelf in wat ik doe en vraag me af hoe ik overkom op anderen
32. Ik zou willen beslissen welk uiterlijk ik wil hebben
33. Ik voel me anders dan hoe anderen me zien
34. Ik schaam me voor mijn lichaam

**BUT-B**

**Kruis met een X het antwoord aan dat uw ervaring op dit moment het best weergeeft.**

**Van mijn lichaam, in het bijzonder, haat ik:**
Antwoordopties: nooit (0), zelden(1), soms(2), vaak(3), heel vaak(4), altijd(5).

1. Lengte
2. De vorm van mijn hoofd
3. De vorm van mijn gezicht
4. Huid
5. Haar
6. Voorhoofd
7. Wenkbrauwen
8. Ogen
9. Neus
10. Lippen
11. Mond
12. Tanden
13. Oren
14. Nek
15. Kin
16. Snor
17. Baard
18. Lichaamshaar
19. Schouders
20. Armen
21. Handen
22. Borstkas
23. Borsten
24. Maag
25. Buik
26. Geslachtsdelen
27. Billen
28. Heupen
29. Dijen
30. Knieën
31. Benen
32. Enkels
33. Voeten
34. Geur
35. Geluiden
36. Zweet
37. Blozen

**Table SA1**

*Mean, SD, skewness and kurtosis and use of response options (%) of BUT-A items (both samples)*

|  |  |  |  |  | Response option | | | | | |
| --- | --- | --- | --- | --- | --- | --- | --- | --- | --- | --- |
| Item | *M* | *SD* | skewness | kurtosis | 0 | 1 | 2 | 3 | 4 | 5 |
| 1 | 2.21 | 1.22 | 0.15 | -0.57 | 7 | 24 | 28 | 27 | 12 | 3 |
| 2 | 1.71 | 1.51 | 0.57 | -0.73 | 28 | 24 | 19 | 14 | 10 | 5 |
| 3 | 2.20 | 1.38 | 0.17 | -0.63 | 13 | 16 | 32 | 20 | 13 | 6 |
| 4 | 2.24 | 1.48 | 0.10 | -0.94 | 15 | 18 | 24 | 21 | 16 | 7 |
| 5 | 1.40 | 1.46 | 0.83 | -0.30 | 37 | 22 | 18 | 10 | 8 | 4 |
| 6 | 2.16 | 1.64 | 0.16 | -1.15 | 23 | 16 | 18 | 20 | 14 | 10 |
| 7 | 1.06 | 1.25 | 1.17 | 0.88 | 46 | 22 | 20 | 7 | 3 | 2 |
| 8 | 0.89 | 1.19 | **1.43** | **1.60** | **52** | 23 | 15 | 6 | 3 | 2 |
| 9 | 1.91 | 1.69 | 0.45 | -1.01 | 29 | 17 | 19 | 14 | 10 | 11 |
| 10 | 2.43 | 1.58 | 0.01 | -1.04 | 16 | 14 | 22 | 21 | 15 | 12 |
| 11 | 1.52 | 1.44 | 0.64 | -0.59 | 33 | 22 | 19 | 14 | 8 | 3 |
| 12 | 2.06 | 1.62 | 0.30 | -1.06 | 23 | 19 | 19 | 18 | 12 | 10 |
| 13 | 0.66 | 0.99 | **1.63** | **2.39** | **60** | 22 | 12 | 3 | 2 | 0 |
| 14 | 1.32 | 1.41 | 0.80 | -0.35 | 41 | 19 | 19 | 12 | 7 | 3 |
| 15 | 0.90 | 1.21 | **1.46** | **1.69** | **53** | 22 | 15 | 5 | 3 | 2 |
| 16 | 0.64 | 1.01 | **1.73** | **2.94** | **63** | 19 | 12 | 4 | 1 | 1 |
| 17 | 1.15 | 1.30 | 0.97 | 0.11 | 44 | 21 | 19 | 9 | 5 | 2 |
| 18 | 2.07 | 1.80 | 0.25 | -1.32 | 31 | 11 | 16 | 15 | 13 | 13 |
| 19 | 0.97 | 1.21 | 1.21 | 0.88 | 50 | 21 | 19 | 6 | 4 | 1 |
| 20 | 1.87 | 1.54 | 0.36 | -0.89 | 27 | 18 | 19 | 22 | 8 | 7 |
| 21 | 1.80 | 1.75 | 0.53 | -1.04 | 35 | 15 | 16 | 13 | 9 | 11 |
| 22 | 2.44 | 1.45 | 0.09 | -0.77 | 11 | 15 | 28 | 23 | 13 | 11 |
| 23 | 2.33 | 1.57 | 0.11 | -1.02 | 16 | 17 | 22 | 20 | 14 | 11 |
| 24 | 0.94 | 1.30 | **1.39** | **1.20** | **55** | 17 | 15 | 7 | 3 | 3 |
| 25 | 0.87 | 1.24 | **1.53** | **1.84** | **56** | 19 | 15 | 5 | 2 | 3 |
| 26 | 1.07 | 1.31 | 1.11 | 0.41 | 48 | 20 | 18 | 7 | 5 | 2 |
| 27 | 1.81 | 1.56 | 0.40 | -0.99 | 29 | 17 | 21 | 15 | 13 | 5 |
| 28 | 1.04 | 1.34 | 1.25 | 0.73 | 50 | 19 | 17 | 6 | 5 | 3 |
| 29 | 0.78 | 1.22 | **1.71** | **2.35** | **61** | 17 | 12 | 4 | 4 | 2 |
| 30 | 1.24 | 1.44 | 1.05 | 0.18 | 44 | 20 | 18 | 9 | 5 | 4 |
| 31 | 2.41 | 1.54 | -0.11 | -0.97 | 17 | 10 | 21 | 25 | 17 | 9 |
| 32 | 2.66 | 1.60 | -0.14 | -1.03 | 13 | 11 | 20 | 22 | 16 | 17 |
| 33 | 2.19 | 1.54 | 0.09 | -1.03 | 20 | 15 | 22 | 22 | 15 | 7 |
| 34 | 1.86 | 1.57 | 0.53 | -0.76 | 25 | 22 | 23 | 13 | 9 | 9 |

*M* = Mean; *SD* = Standard deviation; Response option = Percentage of responses on response option.

**Table SA2**

*Mean, SD, skewness and kurtosis and use of response options (%) of BUT-B items (both samples)*

|  |  |  |  |  | Response option | | | | | |
| --- | --- | --- | --- | --- | --- | --- | --- | --- | --- | --- |
| Item | *M* | *SD* | skewness | kurtosis | 0 | 1 | 2 | 3 | 4 | 5 |
| 1 | 1.00 | 1.21 | 1.22 | 0.96 | 47 | 24 | 18 | 6 | 4 | 2 |
| 2 | 1.12 | 1.23 | 0.91 | 0.00 | 43 | 23 | 19 | 10 | 4 | 1 |
| 3 | 1.49 | 1.38 | 0.58 | -0.56 | 34 | 19 | 24 | 15 | 6 | 3 |
| 4 | 1.56 | 1.40 | 0.61 | -0.54 | 29 | 24 | 22 | 13 | 9 | 3 |
| 5 | 1.32 | 1.27 | 0.77 | -0.20 | 33 | 28 | 20 | 12 | 5 | 1 |
| 6 | 0.92 | 1.23 | **1.40** | **1.32** | **52** | 23 | 13 | 6 | 4 | 2 |
| 7 | 0.81 | 1.12 | **1.41** | **1.37** | **55** | 22 | 12 | 7 | 2 | 1 |
| 8 | 0.51 | 0.91 | **2.07** | **4.38** | **69** | 18 | 9 | 3 | 2 | 0 |
| 9 | 1.31 | 1.42 | 0.91 | -0.09 | 41 | 21 | 19 | 11 | 6 | 4 |
| 10 | 0.92 | 1.20 | **1.30** | **1.07** | **52** | 20 | 17 | 6 | 4 | 1 |
| 11 | 0.79 | 1.14 | **1.48** | **1.53** | **58** | 19 | 14 | 4 | 4 | 1 |
| 12 | 1.37 | 1.34 | 0.80 | -0.13 | 35 | 24 | 24 | 9 | 7 | 2 |
| 13 | 0.40 | 0.87 | **2.40** | **5.65** | **77** | 11 | 7 | 3 | 1 | 0 |
| 14 | 0.59 | 1.06 | **1.90** | **3.15** | **69** | 13 | 10 | 5 | 2 | 1 |
| 15 | 1.04 | 1.38 | 1.21 | 0.53 | **53** | 15 | 15 | 9 | 4 | 3 |
| 16 | 0.46 | 1.00 | **2.48** | **6.11** | 76 | 10 | 7 | 3 | 1 | 1 |
| 17 | 0.20 | 0.69 | **4.11** | **18.37** | **89** | 5 | 2 | 2 | 0 | 0 |
| 18 | 1.49 | 1.44 | 0.69 | -0.43 | 35 | 19 | 24 | 12 | 7 | 4 |
| 19 | 0.89 | 1.27 | 1.31 | 0.75 | 58 | 16 | 13 | 8 | 4 | 1 |
| 20 | 1.37 | 1.46 | 0.75 | -0.45 | 42 | 14 | 22 | 13 | 6 | 4 |
| 21 | 0.79 | 1.16 | **1.56** | **1.96** | **58** | 19 | 13 | 6 | 2 | 2 |
| 22 | 0.92 | 1.33 | 1.35 | 0.80 | 59 | 15 | 12 | 8 | 5 | 2 |
| 23 | 1.62 | 1.57 | 0.62 | -0.72 | 35 | 17 | 21 | 12 | 9 | 6 |
| 24 | 1.37 | 1.63 | 0.84 | -0.62 | 48 | 13 | 13 | 11 | 9 | 6 |
| 25 | 2.72 | 1.69 | -0.16 | -1.16 | 15 | 10 | 21 | 17 | 17 | 20 |
| 26 | 1.14 | 1.36 | 1.22 | 0.81 | 45 | 23 | 18 | 7 | 4 | 4 |
| 27 | 1.58 | 1.48 | 0.65 | -0.44 | 33 | 17 | 25 | 13 | 6 | 6 |
| 28 | 1.55 | 1.57 | 0.65 | -0.77 | 38 | 17 | 17 | 14 | 9 | 5 |
| 29 | 1.71 | 1.7 | 0.53 | -1.03 | 38 | 13 | 16 | 14 | 10 | 8 |
| 30 | 0.77 | 1.22 | **1.72** | **2.34** | **61** | 18 | 11 | 5 | 3 | 2 |
| 31 | 1.63 | 1.64 | 0.63 | -0.82 | 37 | 15 | 19 | 12 | 10 | 7 |
| 32 | 0.44 | 0.96 | **2.71** | **7.73** | **76** | 12 | 6 | 2 | 1 | 1 |
| 33 | 0.91 | 1.31 | **1.47** | **1.43** | **56** | 18 | 13 | 7 | 3 | 3 |
| 34 | 0.97 | 1.15 | 1.16 | 0.89 | 47 | 24 | 19 | 6 | 3 | 1 |
| 35 | 0.77 | 1.14 | **1.47** | **1.54** | **60** | 16 | 14 | 7 | 2 | 1 |
| 36 | 1.64 | 1.53 | 0.66 | -0.58 | 31 | 21 | 22 | 12 | 8 | 6 |
| 37 | 1.13 | 1.46 | 1.16 | 0.29 | 51 | 16 | 15 | 8 | 6 | 4 |

*M* = Mean; *SD* = Standard deviation; Response option = Percentage of responses on response option.

**Table SA3**

*Scale properties (Means, SD, skewness, kurtosis, and reliability indices) of BUT scales (both samples).*

| BUT-A | *M* | *SD* | Skew | Kurt | ω-h | *α* | ω-tot | Mean *r* | *N* items |
| --- | --- | --- | --- | --- | --- | --- | --- | --- | --- |
| General Severity | 1.61 | 1.05 | 0.34 | -0.68 | .82 | .97 | .98 | .54 | 34 |
| Weight phobia | 2.05 | 1.28 | 0.12 | -0.96 | .85 | .91 | .93 | .62 | 8 |
| Body image concern | 1.79 | 1.20 | 0.36 | -0.71 | .86 | .94 | .95 | .63 | 9 |
| Avoidance | 1.16 | 1.06 | 0.82 | -0.01 | .76 | .89 | .91 | .56 | 6 |
| Compulsive self-monitoring | 1.71 | 1.08 | 0.32 | -0.65 | .68 | .82 | .84 | .49 | 5 |
| Depersonalization | 1.16 | 1.11 | 1.00 | 0.49 | .83 | .91 | .92 | .58 | 6 |
| BUT-B |  |  |  |  |  |  |  |  |  |
| Mouth | 0.95 | 0.83 | 0.95 | 0.39 | .65 | .79 | .83 | .39 | 6 |
| Face shape | 0.93 | 0.83 | 0.93 | 0.43 | .57 | .77 | .82 | .34 | 6 |
| Thighs | 1.62 | 1.23 | 0.50 | -0.63 | .70 | .84 | .88 | .51 | 5 |
| Legs | 0.95 | 0.86 | 1.11 | 1.21 | .55 | .71 | .77 | .35 | 5 |
| Arms | 1.19 | 1.04 | 0.89 | 0.17 | .66 | .80 | .83 | .45 | 5 |
| Moustache | 0.72 | 0.82 | 1.81 | 4.37 | .63 | .67 | .71 | .47 | 3 |
| Skin | 1.44 | 1.08 | 0.50 | -0.38 | .00 | .46 | .46 | .30 | 2 |
| Blushing | 1.22 | 0.97 | 0.79 | 0.23 | .59 | .76 | .81 | .37 | 5 |

Mean *r* = mean correlation among items; BUT = body uneasiness test; *α* = Cronbach’s alfa; ω -h = McDonald’s omega-h (hierarchical estimate of the general factor saturation of a scale); ω -tot = omega-total (a model based estimate of the total reliability of a scale); *N* items = number of items of a scale.

*Only two items, no w-h computed.

**Table SA4**

*Comparison of scores of a clinical (n = 436/434) and a community sample (n = 218/217)*

|  | All, *N* = 654 | | Clinical sample, *n* = 436 | | Community sample, *n* = 218 | |  |  |  |  |
| --- | --- | --- | --- | --- | --- | --- | --- | --- | --- | --- |
| Scale = BUT-A | *M* | *SD* | *M* | *SD* | *M* | *SD* | *t* | *p* | *d* | 95%CI |
| General Severity | 1.61 | 1.05 | 2.05 | 0.9 | 0.74 | 0.75 | 19.59 | .0001 | 1.53 | [1.34, 1.71] |
| Weight phobia | 2.05 | 1.28 | 2.56 | 1.07 | 1.02 | 1 | 18.05 | .0001 | 1.47 | [1.28, 1.65] |
| Body image concern | 1.79 | 1.2 | 2.25 | 1.07 | 0.87 | 0.87 | 17.66 | .0001 | 1.37 | [1.19, 1.55] |
| Avoidance | 1.16 | 1.06 | 1.54 | 1.01 | 0.42 | 0.71 | 16.33 | .0001 | 1.21 | [1.04, 1.39] |
| Compulsive self-monitoring | 1.71 | 1.08 | 2.13 | 0.96 | 0.87 | 0.78 | 18.05 | .0001 | 1.40 | [1.22, 1.58] |
| Depersonalization | 1.16 | 1.11 | 1.58 | 1.06 | 0.34 | 0.63 | 18.57 | .0001 | 1.31 | [1.13, 1.49] |
|  | All, *N* = 651 | | Clinical sample, *n* = 434 | | Community sample, *n* = 217 | |  |  |  |  |
| BUT-B | *M* | *SD* | *M* | *SD* | *M* | *SD* | *t* | *p* | *d* | 95%CI |
| Mouth | 0.95 | 0.83 | 1.17 | 0.81 | 0.51 | 0.7 | 10.81 | .0001 | 0.86 | [0.69, 1.03] |
| Face shape | 0.93 | 0.83 | 1.16 | 0.81 | 0.45 | 0.64 | 12.19 | .0001 | 0.94 | [0.77, 1.11] |
| Thighs | 1.62 | 1.23 | 2.06 | 1.15 | 0.74 | 0.84 | 16.66 | .0001 | 1.25 | [1.07, 1.43] |
| Legs | 0.95 | 0.86 | 1.22 | 0.88 | 0.41 | 0.49 | 15.07 | .0001 | 1.05 | [0.88, 1.23] |
| Arms | 1.19 | 1.04 | 1.51 | 1.01 | 0.55 | 0.77 | 13.45 | .0001 | 1.03 | [0.85, 1.20] |
| Moustache | 0.72 | 0.82 | 0.86 | 0.87 | 0.42 | 0.61 | 7.49 | .0001 | 0.55 | [0.39, 0.72] |
| Skin | 1.44 | 1.08 | 1.7 | 1.04 | 0.92 | 0.96 | 9.18 | .0001 | 0.76 | [0.59, 0.93] |
| Blushing | 1.22 | 0.97 | 1.52 | 0.93 | 0.62 | 0.74 | 13.36 | .0001 | 1.03 | [0.86, 1.20] |

BUT = body uneasiness test; *M* = Mean; *SD* = Standard deviation.

| **Table SA5** |  |  |
| --- | --- | --- |
| *Item Rest Correlations of the Body Uneasiness Test in a clinical sample (n = 436/434)* | | |
|  | BUT-A | BUT-B |
| Item | IRC | IRC |
| BUT 01 | **.16** | **.24** |
| BUT 02 | .55 | .46 |
| BUT 03 | .56 | .53 |
| BUT 04 | .72 | .34 |
| BUT 05 | .62 | .36 |
| BUT 06 | .61 | .33 |
| BUT 07 | .59 | .33 |
| BUT 08 | .69 | .47 |
| BUT 09 | .72 | .39 |
| BUT 10 | .72 | .45 |
| BUT 11 | .54 | .47 |
| BUT 12 | .72 | .35 |
| BUT 13 | .64 | .31 |
| BUT 14 | .69 | .46 |
| BUT 15 | .60 | .51 |
| BUT 16 | .62 | **.24** |
| BUT 17 | .36 | **.06** |
| BUT 18 | .72 | .33 |
| BUT 19 | .60 | .48 |
| BUT 20 | .54 | .56 |
| BUT 21 | .64 | .54 |
| BUT 22 | .77 | .55 |
| BUT 23 | .79 | .49 |
| BUT 24 | .40 | .53 |
| BUT 25 | .73 | .49 |
| BUT 26 | .74 | .48 |
| BUT 27 | .64 | .58 |
| BUT 28 | .72 | .58 |
| BUT 29 | .61 | .60 |
| BUT 30 | .66 | .51 |
| BUT 31 | .58 | .56 |
| BUT 32 | .66 | .42 |
| BUT 33 | .73 | .50 |
| BUT 34 | .80 | .43 |
| BUT 35 |  | .50 |
| BUT 36 |  | .38 |
| BUT 37 |  | .36 |
| *Note.* Bold values were inadequate (< .30).  IRC = item rest correlation, BUT = body uneasiness test. | | |

**Table SA6**

*Means (and SD) for genders and t-test results.*

|  | Males (*n* = 112) | | Females (*n* = 541) | |  |  |  |  |
| --- | --- | --- | --- | --- | --- | --- | --- | --- |
| BUT-A | *M* | *SD* | *M* | *SD* | *t* | *p <* | *d* | 95%CI |
| Gen. Severity | 0.84 | 0.77 | 1.77 | 1.04 | -10.87 | .001 | 0.93 | [0.72, 1.14] |
| Weight phobia | 1.08 | 0.98 | 2.25 | 1.24 | -11.02 | .001 | 0.98 | [0.77, 1.19] |
| Body image concern | 1.07 | 0.99 | 1.94 | 1.19 | -8.27 | .001 | 0.76 | [0.55, 0.97] |
| Avoidance | 0.44 | 0.63 | 1.32 | 1.07 | -11.72 | .001 | 0.87 | [0.66, 1.08] |
| Compulsive self-monitoring | 1.00 | 0.92 | 1.86 | 1.05 | -8.87 | .001 | 0.84 | [0.63, 1.05] |
| Depersonalization | 0.45 | 0.67 | 1.31 | 1.12 | -10.84 | .001 | 0.82 | [0.61, 1.02] |
| BUT-B | Males (*n* = 112) | | Females (*n* = 538) | |  |  |  |  |
|  | *M* | *SD* | *M* | *SD* | *t* | *p <* | *d* | 95%CI |
| Mouth | 0.51 | 0.68 | 1.04 | 0.83 | -7.29 | .001 | 0.66 | [0.45, 0.87] |
| Face shape | 0.49 | 0.62 | 1.02 | 0.84 | -7.74 | .001 | 0.66 | [0.45, 0.87] |
| Thighs | 0.68 | 0.75 | 1.82 | 1.22 | -13.02 | .001 | 1.00 | [0.79, 1.21] |
| Legs | 0.47 | 0.54 | 1.06 | 0.88 | -9.17 | .001 | 0.70 | [0.49, 0.91] |
| Arms | 0.71 | 0.86 | 1.29 | 1.04 | -6.31 | .001 | 0.57 | [0.37, 0.78] |
| Moustache | 0.60 | 0.87 | 0.74 | 0.81 | -1.62 | .105 | 0.17 | [0.04, 0.37] |
| Skin | 0.81 | 0.82 | 1.58 | 1.08 | -8.49 | .001 | 0.74 | [0.53, 0.95] |
| Blushing | 0.67 | 0.75 | 1.33 | 0.97 | -8.03 | .001 | 0.70 | [0.50, 0.91] |

BUT = body uneasiness test; *M* = Mean; *SD* = Standard deviation.

**Table SA7**

*Means (and SD) for age groups and ANOVA results.*

|  | 16-20 (*n* = 246) | | 21-23 (*n* = 192) | | 24-72 (*n* = 216) | |  |  |  |  |
| --- | --- | --- | --- | --- | --- | --- | --- | --- | --- | --- |
| BUT-A | *M* | *SD* | *M* | *SD* | *M* | *SD* | *F* | *p <* | *eta^2^* | Tukey HSD |
| General Severity | 1.84 | 0.88 | 1.44 | 1 | 1.5 | 1.23 | 11.75 | .001 | 0.030 | 1>2, 1>3 |
| Weight phobia | 2.36 | 1.11 | 1.89 | 1.21 | 1.84 | 1.44 | 13.40 | .001 | 0.036 | 1>2, 1>3 |
| Body image concern | 2.02 | 1.04 | 1.58 | 1.15 | 1.73 | 1.37 | 9.19 | .001 | 0.024 | 1>2, 1>3 |
| Avoidance | 1.26 | 0.89 | 0.99 | 0.98 | 1.21 | 1.28 | 4.85 | .008 | 0.012 | 1>2 |
| Compulsive self-monitoring | 2.13 | 0.98 | 1.6 | 1.04 | 1.33 | 1.06 | 36.70 | .001 | 0.101 | 1>2, 1>3, 2>3 |
| Depersonalization | 1.3 | 0.95 | 0.99 | 0.99 | 1.17 | 1.33 | 5.26 | .006 | 0.013 | 1>2 |
| BUT-B | 16-20 (*n* = 244) | | 21-23 (*n* = 192) | | 24-72 (*n* = 215) | |  |  |  |  |
|  | *M* | *SD* | *M* | *SD* | *M* | *SD* | *F* | *p <* | *eta^2^* | Tukey HSD |
| Mouth | 1.16 | 0.8 | 0.91 | 0.87 | 0.74 | 0.78 | 15.04 | .001 | 0.044 | 1>2, 1>3 |
| Face shape | 1.11 | 0.79 | 0.84 | 0.76 | 0.8 | 0.89 | 10.10 | .001 | 0.030 | 1>2, 1>3 |
| Thighs | 1.81 | 1.08 | 1.4 | 1.12 | 1.61 | 1.43 | 7.68 | .001 | 0.019 | 1>2 |
| Legs | 1.09 | 0.81 | 0.85 | 0.83 | 0.89 | 0.92 | 4.90 | .008 | 0.015 | 1>2, 1>3 |
| Arms | 1.36 | 0.94 | 1.14 | 1.05 | 1.05 | 1.11 | 5.53 | .004 | 0.017 | 1>3 |
| Moustache | 0.83 | 0.78 | 0.68 | 0.87 | 0.63 | 0.82 | 3.58 | .028 | 0.011 | 1>3 |
| Skin | 1.7 | 1.05 | 1.38 | 1.02 | 1.2 | 1.1 | 12.85 | .001 | 0.038 | 1>2, 1>3 |
| Blushing | 1.16 | 0.8 | 0.91 | 0.87 | 0.74 | 0.78 | 15.04 | .001 | 0.044 | 1>2, 1>3 |

BUT = body uneasiness test; *M* = Mean; *SD* = Standard deviation.

| **Table SB1** | | | | | | | | | | | |  |  |
| --- | --- | --- | --- | --- | --- | --- | --- | --- | --- | --- | --- | --- | --- |
| *Factor loadings of items for the five-factor model of BUT-A items.* | | | | | | | | | | | |  |  |
| Weight Phobia | | Body Image Concerns | | Avoidance | | Compulsive self-monitoring | | | Depersonalization | | | |  |
| BUT09 | .85 | BUT03 | .71 | BUT05 | .86 | | BUT01 | .41 | | BUT02 | .19 | | |
| BUT10 | .88 | BUT04 | .88 | BUT08 | .89 | | BUT11 | .79 | | BUT07 | .82 | | |
| BUT18 | .86 | BUT06 | .82 | BUT13 | .87 | | BUT17 | .65 | | BUT14 | .86 | | |
| BUT21 | .79 | BUT12 | .86 | BUT16 | .83 | | BUT20 | .83 | | BUT26 | .91 | | |
| BUT24 | .57 | BUT15 | .79 | BUT19 | .86 | | BUT27 | .88 | | BUT28 | .89 | | |
| BUT31 | .76 | BUT22 | .93 | BUT30 | .88 | |  |  | | BUT29 | .21 | | |
| BUT32 | .85 | BUT23 | .93 |  |  | |  |  | |  |  | | |
| BUT33 | .88 | BUT25 | .88 |  |  | |  |  | |  |  | | |
|  |  | BUT34 | .92 |  |  | |  |  | |  |  | | |
| BUT = Body Uneasiness Test. | | | | | | | | | | | |  |  |

| **Table SB2** | | | | | | | | | | | | | | | |
| --- | --- | --- | --- | --- | --- | --- | --- | --- | --- | --- | --- | --- | --- | --- | --- |
| *Factor loadings of items for the eight-factor model of BUT-B items.* | | | | | | | | | | | | | | | |
| I |  | II |  | III |  | IV |  | V |  | VI |  | VII |  | VIII |  |
| Mouth | 0.87 | Face Shape | 0.86 | Thighs | 0.88 | Legs | 0.80 | Arms | 0.81 | Hairs | 0.95 | Skin | 0.63 | Buttocks | 0.86 |
| Lips | 0.83 | Head Shape | 0.79 | Hips | 0.85 | Ankles | 0.80 | Breasts | 0.71 | Moustache | 0.74 | Hair | 0.57 | Noises | 0.72 |
| Eyes | 0.76 | Chin | 0.76 | Abdomen | 0.78 | Feet | 0.73 | Chest | 0.68 | Beard | 0.34 |  |  | Odours | 0.69 |
| Nose | 0.69 | Neck | 0.72 | Stomach | 0.76 | Hands | 0.67 | Shoulders | 0.68 |  |  |  |  | Sweating | 0.66 |
| Teeth | 0.64 | Forehead | 0.64 | Knees | 0.72 | Stature | 0.66 | Genitals | 0.41 |  |  |  |  | Blushing | 0.60 |
| Eyebrow | 0.63 | Ears | 0.59 |  |  |  |  |  |  |  |  |  |  |  |  |
| BUT = Body Uneasiness Test, I = mouth, II = face shape, III = thighs, IV = legs, V = arms, VI = moustache, VII = skin, VIII = blushing. | | | | | | | | | | | | | | | |

**Table SC1**

*Cross-Walk Table from Raw Scores to T- and PR-scores for General Severity Index*

| RS | T^1^ | PR_n^2^ | PR_cl^3^ |  | RS | T^1^ | PR_n^2^ | PR_cl^3^ |
| --- | --- | --- | --- | --- | --- | --- | --- | --- |
| 0.00 | 30.0 | 4 | - |  | 1.50 | 61.1 | 87 | 29 |
| 0.03 | 35.6 | 7 | - |  | 1.53 | 61.3 | 87 | 30 |
| 0.06 | 37.7 | 10 | 2 |  | 1.56 | 61.6 | - | 31 |
| 0.09 | 39.2 | 14 | - |  | 1.59 | 61.8 | - | 32 |
| 0.12 | 40.5 | 17 | - |  | 1.62 | 62.1 | 89 | 33 |
| 0.15 | 41.6 | 20 | 2 |  | 1.65 | 62.3 | 89 | 35 |
| 0.18 | 42.6 | 22 | 2 |  | 1.68 | 62.5 | 90 | 36 |
| 0.21 | 43.5 | 25 | 2 |  | 1.71 | 62.8 | 90 | 37 |
| 0.24 | 44.3 | 28 | 2 |  | 1.74 | 63.0 | - | 38 |
| 0.26 | 44.8 | 30 | 2 |  | 1.76 | 63.2 | - | 39 |
| 0.29 | 45.5 | 32 | - |  | 1.79 | 63.4 | 91 | 40 |
| 0.32 | 46.2 | 35 | - |  | 1.82 | 63.6 | - | 42 |
| 0.35 | 46.9 | 38 | 3 |  | 1.85 | 63.8 | - | 43 |
| 0.38 | 47.5 | 40 | 3 |  | 1.88 | 64.0 | - | 44 |
| 0.41 | 48.0 | 42 | 3 |  | 1.91 | 64.3 | 92 | 46 |
| 0.44 | 48.6 | 44 | 3 |  | 1.94 | 64.5 | - | 47 |
| 0.47 | 49.1 | 47 | 3 |  | 1.97 | 64.7 | 93 | 48 |
| 0.50 | 49.7 | 49 | 4 |  | 2.00 | 64.9 | - | 49 |
| 0.53 | 50.2 | 51 | 4 |  | 2.03 | 65.1 | 93 | 51 |
| 0.56 | 50.6 | 53 | 4 |  | 2.06 | 65.3 | - | 52 |
| 0.59 | 51.1 | 54 | 5 |  | 2.09 | 65.5 | - | 53 |
| 0.62 | 51.5 | 56 | 5 |  | 2.12 | 65.7 | 94 | 54 |
| 0.65 | 52.0 | 58 | 6 |  | 2.15 | 65.9 | - | 56 |
| 0.68 | 52.4 | 60 | 6 |  | 2.18 | 66.1 | 94 | 57 |
| 0.71 | 52.8 | 61 | 6 |  | 2.21 | 66.3 | - | 58 |
| 0.74 | 53.2 | - | 7 |  | 2.24 | 66.5 | - | 59 |
| 0.76 | 53.5 | 64 | 7 |  | 2.26 | 66.6 | 95 | 60 |
| 0.79 | 53.9 | 65 | 8 |  | 2.29 | 66.8 | 95 | 61 |
| 0.82 | 54.2 | 67 | 8 |  | 2.32 | 67.0 | - | 63 |
| 0.85 | 54.6 | 68 | 9 |  | 2.35 | 67.2 | - | 64 |
| 0.88 | 55.0 | 69 | 10 |  | 2.38 | 67.4 | - | 65 |
| 0.91 | 55.3 | 71 | - |  | 2.41 | 67.5 | 96 | 66 |
| 0.94 | 55.7 | 72 | 11 |  | 2.44 | 67.7 | 96 | 67 |
| 0.97 | 56.0 | - | 12 |  | 2.47 | 67.9 | - | 68 |
| 1.00 | 56.3 | 74 | 12 |  | 2.50 | 68.1 | - | 69 |
| 1.03 | 56.7 | 75 | 13 |  | 2.53 | 68.3 | - | 70 |
| 1.06 | 57.0 | 76 | 14 |  | 2.56 | 68.4 | - | 72 |
| 1.09 | 57.3 | 77 | 15 |  | 2.59 | 68.6 | - | 73 |
| 1.12 | 57.6 | 78 | 16 |  | 2.62 | 68.8 | - | 74 |
| 1.15 | 57.9 | 79 | 17 |  | 2.65 | 68.9 | - | 75 |
| 1.18 | 58.2 | 80 | 18 |  | 2.68 | 69.1 | - | 76 |
| 1.21 | 58.5 | - | 18 |  | 2.71 | 69.3 | - | 77 |
| 1.24 | 58.8 | 81 | 19 |  | 2.74 | 69.5 | - | 77 |
| 1.26 | 59.0 | 82 | 20 |  | 2.76 | 69.6 | - | 78 |
| 1.29 | 59.2 | - | 21 |  | 2.79 | 69.7 | - | 79 |
| 1.32 | 59.5 | 83 | 22 |  | 2.82 | 69.9 | - | 80 |
| 1.35 | 59.8 | 84 | 23 |  | 2.85 | 70.1 | - | 81 |
| 1.38 | 60.1 | 85 | 24 |  | 2.88 | 70.2 | - | 82 |
| 1.41 | 60.3 | 85 | 25 |  | 2.91 | 70.4 | - | 82 |
| 1.44 | 60.6 | - | 26 |  | 2.94 | 70.5 | - | 83 |
| 1.47 | 60.8 | 86 | 28 |  | 2.97 | 70.7 | - | 84 |
| 3.00 | 70.8 | - | 85 |  | 3.65 | 74.0 | - | 95 |
| 3.06 | 71.2 | 98 | 86 |  | 3.68 | 74.1 | - | 96 |
| 3.09 | 71.3 | - | 87 |  | 3.74 | 74.4 | - | 96 |
| 3.12 | 71.5 | - | 87 |  | 3.76 | 74.5 | 98 | 96 |
| 3.15 | 71.6 | - | 88 |  | 3.82 | 74.7 | - | 97 |
| 3.18 | 71.8 | - | 89 |  | 3.85 | 74.9 | - | 97 |
| 3.21 | 71.9 | - | 89 |  | 3.91 | 75.1 | - | 97 |
| 3.26 | 72.2 | 98 | 90 |  | 3.94 | 75.3 | - | 98 |
| 3.29 | 72.3 | - | 91 |  | 3.97 | 75.4 | - | 98 |
| 3.32 | 72.5 | - | 91 |  | 4.00 | 75.5 | - | 98 |
| 3.35 | 72.6 | - | 92 |  | 4.03 | 75.6 | - | 98 |
| 3.41 | 72.9 | - | 92 |  | 4.09 | 75.9 | - | 98 |
| 3.47 | 73.2 | - | 93 |  | 4.18 | 76.3 | - | 99 |
| 3.50 | 73.3 | - | 94 |  | 4.26 | 76.6 | - | 99 |
| 3.53 | 73.4 | - | 94 |  | 4.38 | 77.1 | - | 99 |
| 3.56 | 73.6 | - | 94 |  | 4.41 | 77.2 | - | 99 |
| 3.59 | 73.7 | - | 95 |  | 5.00 | 79.4 | - | - |
| 3.62 | 73.9 | 98 | 95 |  |  |  |  |  |

NB: RS = Raw Score; T = T-score; PR_n = Percentile Rank score general population; PR_cl = Percentile Rank score clinical sample.

^1^Formula for population all (ID:17) for RS->TRankit: y=30.042+124.958*(1-exp(-exp(4.685e-01*(ln(x+.0001)-ln(21.742))))); a Weib2 function.

^2^Formula for population all (ID:15) for RS->PR_n: y=-134.288+232.907*(exp(-exp(-1.545*(x+4.201e-01)))); a gompertz2 function.

^3^Formula for clinical all (ID:17) for RS->PR_cl: y=1.824+98.366*(1-exp(-exp(2.527*(ln(x+.0001)-ln(2.356))))); a Weib2 function.

**Table SC2**

*Cross-Walk Table from Raw Scores to T- and PR-scores for Weight phobia*

| RS | T^1^ | PR_n^2^ | PR_cl^3^ |
| --- | --- | --- | --- |
| 0.00 | 36.1 | 9 | 1 |
| 0.12 | 40.9 | 16 | 1 |
| 0.25 | 43.4 | 24 | 1 |
| 0.38 | 45.5 | 32 | 2 |
| 0.50 | 47.1 | 38 | 2 |
| 0.62 | 48.5 | 44 | 3 |
| 0.75 | 49.9 | 50 | 4 |
| 0.88 | 51.2 | 56 | 6 |
| 1.00 | 52.4 | 60 | 7 |
| 1.12 | 53.5 | 64 | 9 |
| 1.25 | 54.6 | 68 | 12 |
| 1.38 | 55.6 | 72 | 15 |
| 1.50 | 56.6 | 75 | 17 |
| 1.62 | 57.5 | 78 | 20 |
| 1.75 | 58.4 | 80 | 24 |
| 1.88 | 59.4 | 83 | 28 |
| 2.00 | 60.2 | 85 | 31 |
| 2.12 | 61.0 | 86 | 35 |
| 2.25 | 61.8 | 88 | 39 |
| 2.38 | 62.6 | 89 | 44 |
| 2.50 | 63.3 | 91 | 48 |
| 2.62 | 64.1 | 92 | 52 |
| 2.75 | 64.8 | 93 | 56 |
| 2.88 | 65.6 | 94 | 60 |
| 3.00 | 66.2 | 94 | 64 |
| 3.12 | 66.9 | 95 | 68 |
| 3.25 | 67.6 | - | 72 |
| 3.38 | 68.3 | - | 76 |
| 3.50 | 68.9 | 96 | 79 |
| 3.62 | 69.5 | 97 | 82 |
| 3.75 | 70.1 | 97 | 85 |
| 3.88 | 70.8 | 97 | 87 |
| 4.00 | 71.4 | - | 90 |
| 4.12 | 71.9 | 98 | 92 |
| 4.25 | 72.5 | 98 | 94 |
| 4.38 | 73.1 | 98 | 96 |
| 4.50 | 73.7 | - | 97 |
| 4.62 | 74.2 | - | 99 |
| 4.75 | 74.8 | - | 100 |
| 4.88 | 75.3 | - | - |
| 5.00 | 75.9 | - | 100 |

NB: RS = Raw Score; T = T-score; PR_n = Percentile Rank score general population; PR_cl = Percentile Rank score clinical sample.

^1^Formula for population all (ID:17) for RS->TRankit: y=36.106+233.685*(1-exp(-exp(5.892e-01*(ln(x+.0001)-ln(86.491))))); a Weib2 function.

^2^Formula for population all (ID:12) for RS->PR_n: y=-180.699+280.192/(1+exp(-1.076*(x+6.836e-01))); a sigm-L4 function.

^3^Formula for clinical all (ID:17) for RS->PR_cl: y=1.262+104.252*(1-exp(-exp(2.473*(ln(x+.0001)-ln(3.092))))); a Weib2 function.

**Table SC3**

*Cross-Walk Table from Raw Scores to T- and PR-scores for*

*Body image concern*

| RS | T^1^ | PR_n^2^ | PR_cl^3^ |  | RS | T^1^ | PR_n^2^ | PR_cl^3^ |
| --- | --- | --- | --- | --- | --- | --- | --- | --- |
| 0.00 | 36.1 | 9 | 0 |  | 2.56 | 66.1 | 94 | 61 |
| 0.11 | 41.1 | 17 | 0 |  | 2.67 | 66.8 | 95 | 65 |
| 0.22 | 43.6 | 25 | 0 |  | 2.78 | 67.5 | - | 68 |
| 0.33 | 45.6 | 32 | 2 |  | 2.89 | 68.1 | 96 | 72 |
| 0.44 | 47.3 | 39 | 4 |  | 3.00 | 68.7 | - | 75 |
| 0.56 | 49.0 | 46 | 5 |  | 3.11 | 69.4 | 97 | 78 |
| 0.67 | 50.4 | 52 | 7 |  | 3.22 | 70.0 | 97 | 81 |
| 0.78 | 51.7 | 57 | 9 |  | 3.33 | 70.6 | 97 | 83 |
| 0.89 | 52.9 | 62 | 12 |  | 3.44 | 71.2 | - | 86 |
| 1.00 | 54.0 | 66 | 14 |  | 3.56 | 71.8 | 98 | 88 |
| 1.11 | 55.1 | 70 | 16 |  | 3.67 | 72.3 | - | 90 |
| 1.22 | 56.2 | 74 | 19 |  | 3.78 | 72.9 | - | 91 |
| 1.33 | 57.1 | 77 | 22 |  | 3.89 | 73.4 | - | 93 |
| 1.44 | 58.1 | 79 | 25 |  | 4.00 | 74.0 | - | 94 |
| 1.56 | 59.1 | 82 | 28 |  | 4.11 | 74.5 | - | 95 |
| 1.67 | 60.0 | 84 | 31 |  | 4.22 | 75.0 | 99 | 96 |
| 1.78 | 60.8 | 86 | 35 |  | 4.33 | 75.5 | - | 97 |
| 1.89 | 61.6 | 88 | 38 |  | 4.44 | 76.0 | - | 97 |
| 2.00 | 62.4 | 89 | 42 |  | 4.56 | 76.6 | - | 98 |
| 2.11 | 63.2 | 90 | 46 |  | 4.67 | 77.0 | 99 | 98 |
| 2.22 | 63.9 | 92 | 49 |  | 4.78 | 77.5 | - | 99 |
| 2.33 | 64.7 | 93 | 53 |  | 4.89 | 78.0 | - | 99 |
| 2.44 | 65.4 | 93 | 57 |  | 5.00 | 78.5 | - | - |

NB: RS = Raw Score; T = T-score; PR_n = Percentile Rank score general population; PR_cl = Percentile Rank score clinical sample.

^1^Formula for population all (ID:17) for RS->TRankit: y=36.121+129.669*(1-exp(-exp(6.074e-01*(ln(x+.0001)-ln(23.049))))); a Weib2 function.

^2^Formula for population all (ID:15) for RS->PR_n: y=-79.452+178.795*(exp(-exp(-1.244*(x+2.786e-01)))); a gompertz2 function.

^3^Formula for clinical all (ID:13) for RS->PR_cl: y=-21.488+122.397/((1+exp(-1.655*(x-2.898)))^3.889e-01); a sigm-L5 function.

**Table SC4**

*Cross-Walk Table from Raw Scores to T- and PR-scores for Avoidance*

|  | all | | |
| --- | --- | --- | --- |
| RS | T^1^ | PR_n^2^ | PR_cl^3^ |
| 0.00 | 42.4 | 22 | 2 |
| 0.17 | 50.7 | 53 | 8 |
| 0.33 | 53.8 | 65 | 13 |
| 0.50 | 56.2 | 73 | 18 |
| 0.67 | 58.1 | 79 | 24 |
| 0.83 | 59.7 | 83 | 29 |
| 1.00 | 61.2 | 87 | 35 |
| 1.17 | 62.5 | 89 | 40 |
| 1.33 | 63.6 | 91 | 46 |
| 1.50 | 64.7 | 93 | 51 |
| 1.67 | 65.7 | 94 | 57 |
| 1.83 | 66.5 | 95 | 62 |
| 2.00 | 67.4 | 96 | 68 |
| 2.17 | 68.2 | 97 | 73 |
| 2.33 | 69.0 | 97 | 78 |
| 2.50 | 69.7 | 98 | 82 |
| 2.67 | 70.4 | - | 86 |
| 2.83 | 71.0 | - | 89 |
| 3.00 | 71.6 | 99 | 92 |
| 3.17 | 72.2 | - | 94 |
| 3.33 | 72.8 | - | 96 |
| 3.50 | 73.3 | - | 97 |
| 3.67 | 73.9 | - | 97 |
| 3.83 | 74.4 | 100 | 98 |
| 4.00 | 74.9 | - | 98 |
| 4.17 | 75.3 | - | 99 |
| 4.33 | 75.8 | 100 | 99 |
| 4.50 | 76.2 | - | 99 |
| 4.67 | 76.6 | 100 | - |
| 4.83 | 77.0 | - | - |
| 5.00 | 77.4 | - | 99 |

NB: RS = Raw Score; T = T-score; PR_n = Percentile Rank score general population; PR_cl = Percentile Rank score clinical sample.

^1^Formula for population all (ID:17) for RS->TRankit: y=42.439+63.579*(1-exp(-exp(5.157e-01*(ln(x+.0001)-ln(7.708))))); a Weib2 function.

^2^Formula for population all (ID:17) for RS->PR_n: y=22.476+78.288*(1-exp(-exp(6.999e-01*(ln(x+.0001)-ln(4.671e-01))))); a Weib2 function.

^3^Formula for clinical all (ID:13) for RS->PR_cl: y=-541.029+640.068/((1+exp(-2.555*(x-2.820)))^2.277e-02); a sigm-L5 function.

**Table SC5**

*Cross-Walk Table from Raw Scores to T- and PR-scores for Compulsive self-monitoring.*

|  | all | | |
| --- | --- | --- | --- |
| RS | T^1^ | PR_n^2^ | PR_cl^3^ |
| 0.0 | 34.8 | 7 | 0 |
| 0.2 | 41.5 | 19 | 1 |
| 0.4 | 45.5 | 32 | 3 |
| 0.6 | 48.7 | 45 | 6 |
| 0.8 | 51.4 | 56 | 9 |
| 1.0 | 53.9 | 65 | 13 |
| 1.2 | 56.1 | 73 | 18 |
| 1.4 | 58.1 | 80 | 23 |
| 1.6 | 60.0 | 85 | 30 |
| 1.8 | 61.8 | 88 | 37 |
| 2.0 | 63.4 | 91 | 45 |
| 2.2 | 65.0 | 93 | 53 |
| 2.4 | 66.4 | 95 | 61 |
| 2.6 | 67.8 | 96 | 69 |
| 2.8 | 69.2 | 96 | 76 |
| 3.0 | 70.4 | - | 81 |
| 3.2 | 71.6 | - | 86 |
| 3.4 | 72.8 | 98 | 90 |
| 3.6 | 73.9 | - | 93 |
| 3.8 | 75.0 | 98 | 95 |
| 4.0 | 76.1 | - | 97 |
| 4.2 | 77.1 | 98 | 98 |
| 4.4 | 78.0 | - | 99 |
| 4.6 | 79.0 | - | 100 |
| 4.8 | 79.9 | - | - |
| 5.0 | 80.8 | - | - |
| NB: RS = Raw Score; T = T-score; PR_n = Percentile Rank score general population; PR_cl = Percentile Rank score clinical sample. | | | |
| ^1^Formula for population all (ID:18) for RS->TRankit: y=537.575-502.781/((1+(x/3.849)^7.247e-01)^1.211e-01); a logis5 function. | | | |
| ^2^Formula for population all (ID:12) for RS->PR_n: y=-52.004+150.268/(1+exp(-1.722*(x-2.586e-01))); a sigm-L4 function. | | | |
| ^3^Formula for clinical all (ID:13) for RS->PR_cl: y=-6.507+107.873/((1+exp(-1.666*(x-2.296)))^7.574e-01); a sigm-L5 function. | | | |

**Table SC6**

*Cross-Walk Table from Raw Scores to T- and PR-scores for Depersonalization*.

|  | all | | |
| --- | --- | --- | --- |
| RS | T^1^ | PR_n^2^ | PR_cl^3^ |
| 0.0 | 43.2 | 25 | 1 |
| 0.2 | 53.0 | - | - |
| 0.4 | 56.5 | - | - |
| 0.6 | 59.0 | - | - |
| 0.8 | 61.0 | - | - |
| 1.0 | 62.7 | 90 | 34 |
| 1.2 | 64.1 | - | - |
| 1.4 | 65.4 | - | - |
| 1.6 | 66.6 | - | - |
| 1.8 | 67.7 | - | - |
| 2.0 | 68.7 | 96 | 70 |
| 2.2 | 69.6 | - | - |
| 2.4 | 70.5 | - | - |
| 2.6 | 71.3 | - | - |
| 2.8 | 72.0 | - | - |
| 3.0 | 72.7 | 99 | 90 |
| 3.2 | 73.4 | - | - |
| 3.4 | 74.1 | - | - |
| 3.6 | 74.7 | - | - |
| 3.8 | 75.3 | - | - |
| 4.0 | 75.9 | - | 97 |
| 4.2 | 76.4 | - | - |
| 4.4 | 77.0 | - | - |
| 4.6 | 77.5 | - | - |
| 4.8 | 78.0 | - | - |
| 5.0 | 78.4 | - | 99 |
| NB: RS = Raw Score; T = T-score; PR_n = Percentile Rank score general population; PR_cl = Percentile Rank score clinical sample. | | | |
| ^1^Formula for population all (ID:18) for RS->TRankit: y=839.887-796.637/((1+(x/2.679)^5.113e-01)^5.222e-02); a logis5 function. | | | |
| ^2^Formula for population all (ID:18) for RS->PR_n: y=106.239-81.239/((1+(x/1.447e-01)^1.198)^6.564e-01); a logis5 function. | | | |
| ^3^Formula for clinical all (ID:12) for RS->PR_cl: y=-21.817+121.469/(1+exp(-1.283*(x-1.126))); a sigm-L4 function. | | | |
